# Spontaneous pneumomediastinum and subcutaneous emphysema in avian influenza A (H5N6) human pneumonia

**DOI:** 10.1002/ccr3.2519

**Published:** 2019-11-11

**Authors:** Xiang Zhang, Jiwen Wang, Qiaojun Zeng, Xiaoyan Wu, Shanping Jiang, Jun Shen

**Affiliations:** ^1^ Department of Radiology Sun Yat‐Sen Memorial Hospital Sun Yat‐Sen University Guangzhou China; ^2^ Department of Pulmonary and Critical Care Medicine Sun Yat‐Sen Memorial Hospital Sun Yat‐Sen University Guangzhou China

**Keywords:** avian influenza, computed tomography, H5N6, pneumomediastinum, pneumonia, subcutaneous emphysema

## Abstract

The pneumomediastinum and subcutaneous emphysema can repetitively occur in human H5N6 virus pneumonia. Prompt treatment of this uncommon complication is important for successful rescue of patients with H5N6 virus pneumonia.

## Graphical abstract

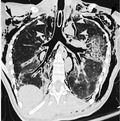

A 22‐year‐old man, with 4‐day high fever, paroxysmal dry cough, and shortness of breath, showed large consolidation in the lower lobe of the right lung on computed tomography (CT) (Figure 1A), which rapidly progressed to bilateral lungs 3 days later (Figure 1B). RT‐PCR assay confirmed the infection of influenza A (H5N6) virus, and other respiratory pathogens were excluded. The patient was transferred to the intensive care unit and treated with mechanical ventilation (positive end‐expiratory pressure [PEEP] = 15 cm H_2_O; peak inspiratory pressure [PIP] = 40 cm H_2_O). After one day, mediastinal and chest wall subcutaneous emphysema occurred as revealed by CT scan (Figure 1C), which disappeared 13 days later on CT images (Figure 1D) after treated with incision of the suprasternal fossa and the left subcutaneous chest wall. A 2‐cm incision of skin and subcutaneous tissue was performed, respectively, on suprasternal fossa (blunt dissection to pretracheal fascia) and the second intercostal space in the middle line of left clavicle (blunt dissection to superficial side or rib) to avoid the cardiac and vascular compression and improve the subcutaneous emphysema. Mechanical ventilation was already withdrawn 3 days after emphysema relief. However, 6 days later, mediastinal and subcutaneous emphysema recurred as shown by CT (Figure 1E). The patient was treated again with an incision of the suprasternal fossa and the subcutaneous chest wall. After 21 days, another CT scan (Figure 1F) demonstrated the disappearance of emphysema. Hence, the patient was diagnosed with the complication of mediastinal and chest wall subcutaneous emphysema and was successfully treated with incision of the suprasternal fossa and the left subcutaneous chest wall.

**Figure 1:**
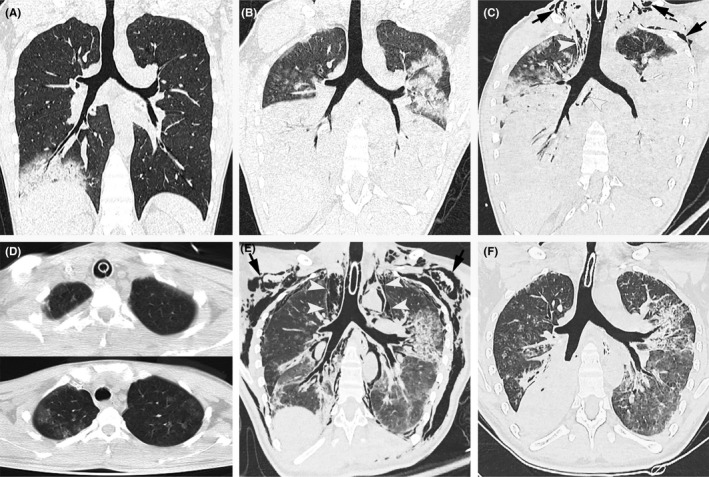
Chest CT in a 22‐year‐old man with avian influenza A (H5N6) pneumonia. A, CT image showed large consolidation in the lower lobe of the right lung on admission. B, CT image showed the rapid progression of the consolidation to bilateral lungs. C, CT image showed the first occurrence of subcutaneous emphysema (arrow) and pneumomediastinum (arrowhead). D, CT images showed the first relief of subcutaneous emphysema and pneumomediastinum. E, CT image showed the second occurrence of subcutaneous emphysema (arrow) and pneumomediastinum (arrowhead). F, CT image showed the second relief of subcutaneous emphysema and pneumomediastinum

The spontaneous pneumomediastinum and subcutaneous emphysema is uncommon in avian influenza A human pneumonia. It occurred in a patient with H1N1 virus pneumonia.^1^ Here, we reported its occurrence in a patient with H5N6 virus pneumonia. In this case, the initial onset of pneumomediastinum and subcutaneous emphysema may be associated with invasive mechanical ventilation, while the secondary onset of pneumomediastinum and subcutaneous emphysema may be associated with pulmonary infection because it occurred spontaneously without any mechanical ventilation or tracheotomy. Hence, spontaneous pneumomediastinum and subcutaneous emphysema should be considered as a potential complication in avian influenza A human pneumonia, though its cause remains unknown.

## CONFLICT OF INTEREST

None declared.

## AUTHOR CONTRIBUTIONS

ZX and XW: captured the images, coconducted the literature review, and cowrote the paper. JW and QZ: treated the patient, coconducted the literature review, and cowrote the paper. SPJ: analyzed the case, coconducted the literature review, and cowrote the paper. JS: cowrote the paper and coordinated and corrected the paper.
